# Retraction Note: A one-year observational cohort study of menstrual cramps and ovulation in healthy, normally ovulating women

**DOI:** 10.1038/s41598-024-79233-1

**Published:** 2024-11-19

**Authors:** Sewon Bann, Azita Goshtasebi, Sonia Shirin, Jerilynn C. Prior

**Affiliations:** 1 MD Internal Medicine Postgraduate Program (2023), University of British Columbia, Vancouver, Canada; 2 Endocrinology and Metabolism, Centre for Menstrual Cycle and Ovulation Research (CeMCOR), University of British Columbia, 2775 Laurel Street, 4th Floor, Vancouver, BC V5Z 1M9 Canada; 3 Division of Endocrinology, Department of Medicine, University of British Columbia, Vancouver, Canada; 4 BC Women’s Health Research Institute, Vancouver, Canada; 5 School of Population and Public Health, University of British Columbia, Vancouver, Canada

Retraction of: *Scientific Reports* 10.1038/s41598-022-08658-3, published online 18 March 2022

The Authors have retracted this Article.

Following publication, the Authors identified that inadvertently 26 cycles that were categorised as ‘anovulatory’ (of a total of 720 cycles) were in fact missing information and should have been excluded. Due to this, the anovulatory cycles that can be analysed are reduced to 19 and the number of women whose data can be used for a direct comparison of ovulatory and anovulatory cycles is decreased to 9. As such, the within-woman analysis that documented no differences in cramp duration, intensity, nor Cramp Score by ovulatory/anovulatory status now lacks statistical power. The Authors therefore lack confidence in the reliability of the main conclusions presented in this study.

All Authors agree to this retraction.

